# Natural Products for Infectious Diseases

**DOI:** 10.1155/2016/9459047

**Published:** 2016-09-22

**Authors:** Kang-Ju Kim, Xiangqian Liu, Takashi Komabayashi, Seung-Il Jeong, Serkan Selli

**Affiliations:** ^1^Wonkwang University School of Dentistry, Iksan, Republic of Korea; ^2^Hunan University of Chinese Medicine School of Pharmacy, Changsha, China; ^3^University of New England College of Dental Medicine, Portland, ME, USA; ^4^Jeonju AgroBio-Materials Institute, Jeonju, Republic of Korea; ^5^University of Cukurova Faculty of Agriculture, Adana, Turkey

Infectious diseases have represented a threat to human lives since the beginning of human existence. Many infectious diseases have been conquered through the discovery of antibiotics and antiviral agents. However, the antibiotic-resistant strains and mutant microorganisms that are now emerging are more powerful than the existing ones. In addition, some existing microorganisms have developed resistance to antibiotics, leading to infections that are more difficult to treat. Moreover, microbial biofilms cannot be treated by antibiotics and can cause chronic infections. Infectious diseases continue to pose a threat to humans, and continued efforts are needed to develop effective treatments.

In recent times, natural products have been as widely used as chemical drugs against clinical diseases. Most chemical drugs that are widely used today were isolated from natural products, and thus natural products will continue to be important raw materials for the development of new drugs. However, since natural products are the byproducts of empirical medicine, they lack scientific validation. Currently, various scientific experiments are being conducted to fill this gap by evaluating the efficacy of natural product.

This special issue includes 7 research articles and 1 review article addressing the efficacies of natural products for treating infectious diseases, such as infection by multidrug-resistant bacteria, viral influenza, coccidiosis, leishmaniasis, infectious septic shock, and biofilm formation. These articles represent pharmacological activity tests, investigation of action mechanisms of natural products, clinical trials with scientific statistical analyses, and phytochemical analyses of bioactive components in medicinal plants, which are important for scientific validation of the use of natural products in alternative and complimentary medicine.

